# Shifting entrepreneurial landscape and development performance of water startups in emerging water markets

**DOI:** 10.1371/journal.pone.0246282

**Published:** 2021-02-04

**Authors:** Peiyuan Liu, Yuxiong Huang, Slav W. Hermanowicz

**Affiliations:** 1 Tsinghua-Berkeley Shenzhen Institute, Tsinghua Shenzhen International Graduate School, Tsinghua University, Shenzhen, China; 2 Department of Civil and Environmental Engineering, University of California, Berkeley, CA, United States of America; China Agricultural University, CHINA

## Abstract

Emerging technologies have driven the rise of many water-related startups and created new opportunities in water markets. The global water crisis could be mitigated by applying innovative technologies, sound water management decisions, and successful business models, and it is essential to better understand the status and future trends of emerging water markets. This study aims to discover shifts in the entrepreneurial landscape and evaluate water startups' development performance for the sustainable development of emerging water markets. We collected and analyzed data including the founding date, service area, service provided, details of funding raised, revenues, and consumer responses on 132 water startups founded between 2008 and 2018 in California, USA. Our results indicated that municipal area dominated the emerging water startup market compared to agricultural and industrial areas, and that many of the services provided shifted from conventional technologies to digital technologies. Though digital water startups' current revenues were relatively low, digital techniques applied in the water industry exhibited the good potential to promote public health and water saving. The development trends and performance of water startups enlighten the technological and commercial revolutions in the emerging water market, and provide guidelines for the decision-making in relevant stakeholders in the scientific, governmental, and industrial communities.

## Introduction

Water is one of the most critical global issues, particularly water accessibility, water quality, and water management. By 2050, global water demand is expected to increase 20 to 30% of current water use [1]. Nearly half of the global population has already been living in areas that experience potential water scarcity at least one month per year, and such scarcity could affect from 4.8 to 5.7 billion people by 2050 [2]. Even worse, it is reported that there was 2000 million kg of sewage and other effluents discharged directly into water bodies without any treatment every day in developing countries [3]. This unmitigated pollution has caused the severe contamination of water sources with pathogens (e.g., bacteria, viruses, protozoa), heavy metals, toxic organic substances, and emerging pollutants (e.g., pharmaceuticals and personal care products (PPCPs), endocrine disrupting compounds (EDCs)), which may further result in public health concern and eco-environmental risks [4]. Water used in agronomy accounts for more than 50% of the water consumption every year, while the agricultural production also causes contamination of water sources [5,6]. There were also various problems in water management, including the lack of planning, distributing, and managing the optimal use of water resources. For example, a global estimated 3.2×10^4^ million m^3^ of water was lost during the water distribution each year [7]. Half of the water losses occur in developing countries, where roughly 45 million m^3^ of water was lost daily through water leakage in the distribution networks, enough to serve nearly 200 million people. The efficiency of water resources management should be improved.

Effective solutions should be provided at different levels to address global water issues, from the micro-level (research and development (R&D) of innovative technology) to the meso-level (commercialization), and to the macro-level (government management). Innovative R&D in the water sector has been studied extensively. For example, nanotechnologies were used for water treatment and purification [8]. Solar-enabled technologies were applied for desalination, detoxification, and disinfection [9]. Another burgeoning topic was the engagement of digital technologies, including sensors for monitoring water quality [10] and big data analytics on water supplies [11]. There were also studies providing strategies for governmental water management, including the adaptive paradigm for water policy management [12] and the Integrated Water Resources Management (IWRM) approach [13]. However, the economic development and population growth now require more water resources globally, and the rate and intensity of climate change is also contributing to water depletion worldwide [14]. Coping with the increasingly severe water crisis can not only depend on the solutions of the micro-level (R&D of innovative technology) and the macro-level (government management). The meso-level (commercialization), such as applications in the commercial water market, needs to be paid more attention to because practical applications could provide water value for the public directly and solve problems efficiently. However, only few studies have addressed the commercial water market, mainly focusing on sustainable business models [15] and water rights transactions [16]. Little attention was paid to water startups in commercial markets. These startups are young companies founded by entrepreneurs to develop a unique product or service. The entrepreneurs have their focus shifted in primary services, target markets, and development performance in the last decade. It is essential to investigate these changes in this entrepreneurial landscape. Water entrepreneurship, which aims to solve significant societal and environmental problems and move beyond an exclusive focus on business pragmatism, has played a vital role in guaranteeing water quantity and water quality, not only for our generation but also for future generations [17]. To promote the sustainable development of the emerging water market, water startups' shifting landscape and development performance should be examined. The outcomes of such examination will undoubtedly guide the decision-making by relevant stakeholders (i.e., entrepreneurs, scientists, policymakers, venture capitalists, and consumers).

**Fig 1:**
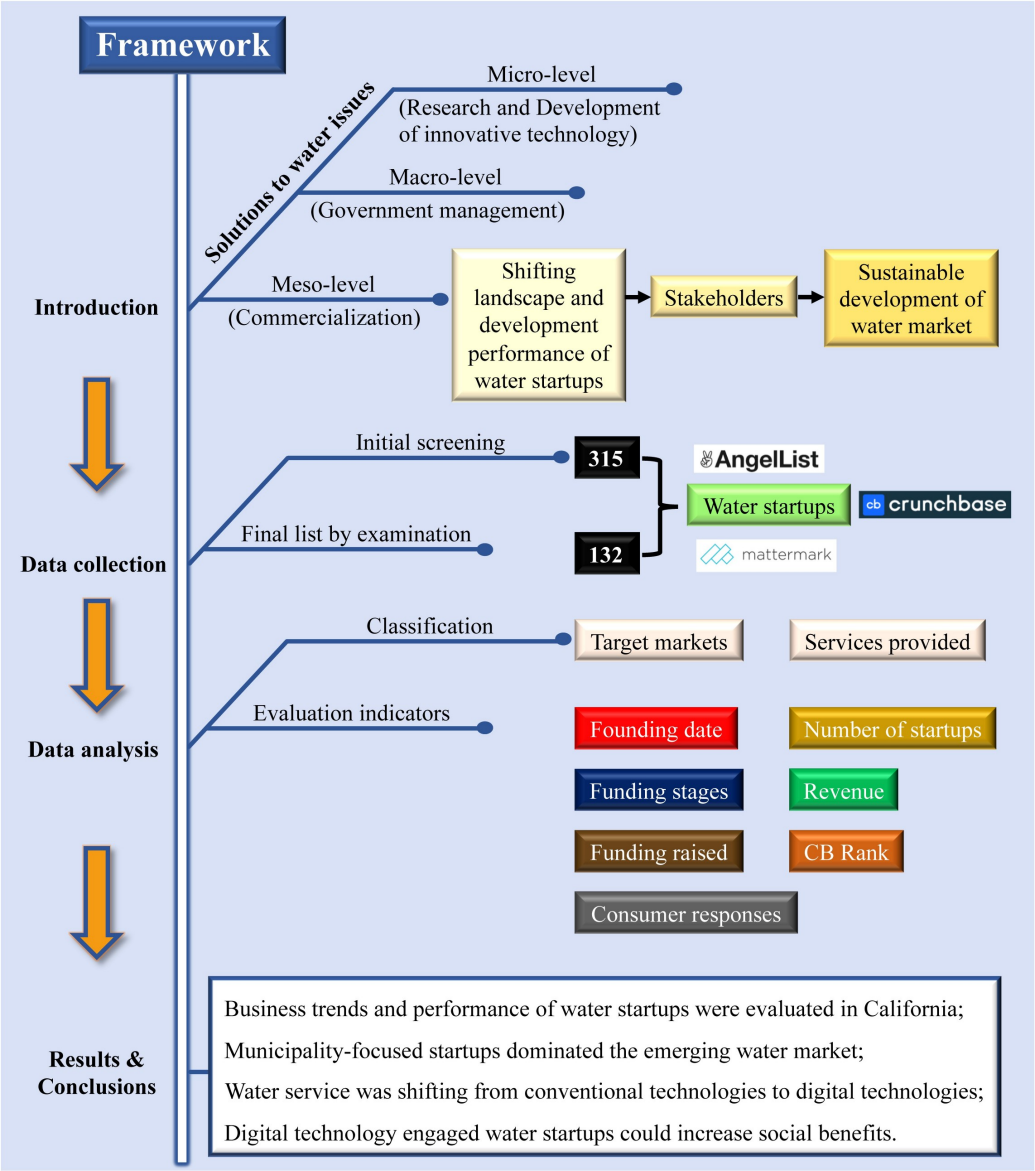
The framework of the study. The search strategy of data collection and the definitions of evaluation indicators are provided in the SI as S1 and S3 Text, respectively.

To investigate the recent changes in the emerging water market, we compiled a dataset covering water startups founded between 2008 and 2018 in California, USA. California is one of the most active enterprise markets globally and has the world’s largest and most productive water system. It could be a representative case study to explore changing trends and make future perspectives. From 2008 to 2018, California was faced with a continuous drought that the mean value of the Palmer Drought Severity Index (PDSI) was at -2.26 in the period [18]. The continuous drought state had resulted in negative effects on water supply in California. Meanwhile, the continuous growing population and urbanization increase the water use demand, which is projected a 4.1% increase by 2062 according to the US Geological Survey and the Nature Conservancy (TNC) [19]. The huge gap between water supply and demand required measures and actions to mitigate the water crisis. Although California carried out mandatory measures, such as the first urban water use law (2011) and Sustainable Groundwater Management as the first groundwater management legislation in the state’s history (2014) [19], which only partially relieve water use stress. To better solve the water issues, more technological innovations should be emerged to improve water quality, reduce water use, and increase water management efficiency sustainably. The objective of this study is to discover shifts in the entrepreneurial landscape and evaluate the development performance of water startups for the sustainable development of emerging water markets. The following shows the framework flow process for this study (Fig 1). It started with scope definition followed by data collection and analysis leading to conclusions with details in the subsequent sections.

## Methods

### Data collection

Detailed information about the water startups was collected from Crunchbase, a leading database for professionals to acquire business information about innovative companies worldwide, including general information, financial information, and marketplace information [20]. The search was limited by the following criteria: headquarters in California, water-related business, and the founding date between 1/1/2008 and 12/31/2018. The search in Crunchbase yielded a total of 269 water startups in February 2019. Another two databases, AngelList and Mattermark [21,22], were used to cross-check to ensure comprehensive data collection, adding another 46 water startups. Details of the search strategy are in S1 Text of Supporting Information (SI).

The data on the 315 water startups were examined to verify the locations (California), founding dates (between 2008 and 2018), and specific services provided (improving water quality, saving water quantity, or increasing water management efficiency directly and indirectly) based on information available on the startups’ official websites and Crunchbase. Eventually, the list was refined to 132 qualified water startups, as shown in S1 Dataset.

### Data analysis

These 132 startups were coded based on target markets and the services provided. Target markets included agriculture, industry, and municipality/household areas. Specifically, water services are provided to improve water efficiency and/or crop yield in agricultural area, while the services provided to industrial and municipality/household areas are mostly to monitor water leaking, improve water quality, etc. Note that some startups provide services to more than one area/market. Services provided by the water startups included design, consulting, venture capital (VC)/financing/incubator/accelerator, non-governmental organization (NGO) activities, digital technology, and physical/chemical/biological technology. The specific service contents are listed in S2 Text.

Several representative indicators were selected for further analysis, including target market, service provided, founding date, funding raised (e.g., funding status, total funding amount.), revenue per year, Crunchbase Rank (CB Rank), consumer responses on desktop and mobile webs (e.g., average visits, page views/visit, details are provided in S3 Text). Particularly, the CB Rank is a dynamic ranking for all entities (e.g., companies, organizations) in the Crunchbase dataset that measures the prominence of an entity. The CB Rank is determined by a proprietary algorithm that incorporates total funding amount, page views, follows, funding events, news articles, acquisitions, the number of connections a profile has, the level of community engagement, and other factors [23–25]. A company’s CB Rank is fluid and can rise or decay over time, depending on time-sensitive events. Events such as product launches, funding events, leadership changes, and news affect a company’s CB Rank (Details are explained in S3 Text).

Based on above the indicators, the distributions of water startups among different founding year, target markets, and service areas were investigated with flow analysis and presented in Sankey diagrams. The water startup business performance and market feedback, including the financial situation, consumer responses, and CB Rank were quantitatively analyzed. SankeyMATIC [26] was used to plot Sankey diagrams, and R software was used to plot violin plots.

## Results and discussion

### Shifting landscape of emerging water markets

**Fig 2:**
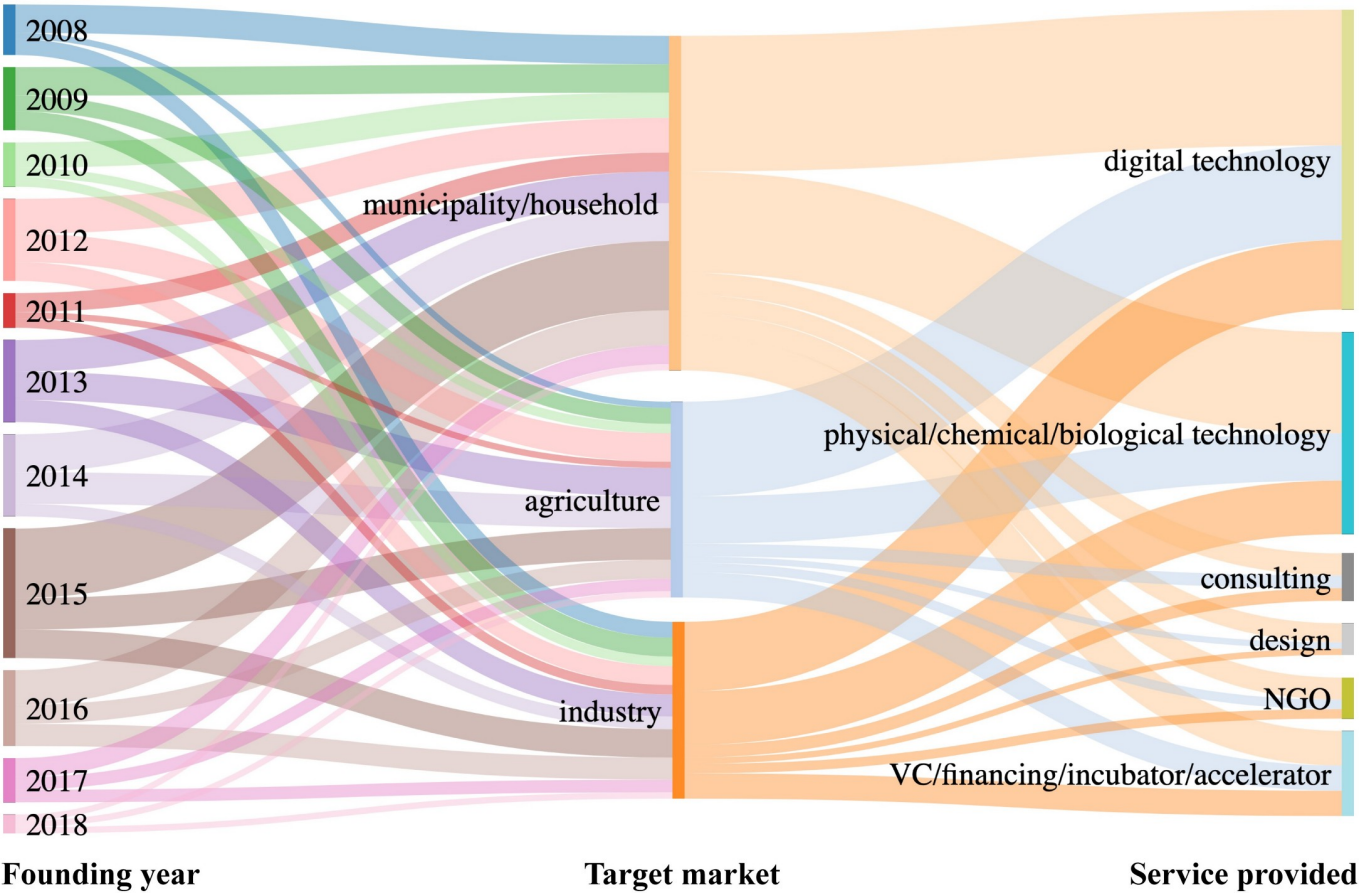
The distributions of water startup numbers among the founding year, target markets, and services.

**Fig 3:**
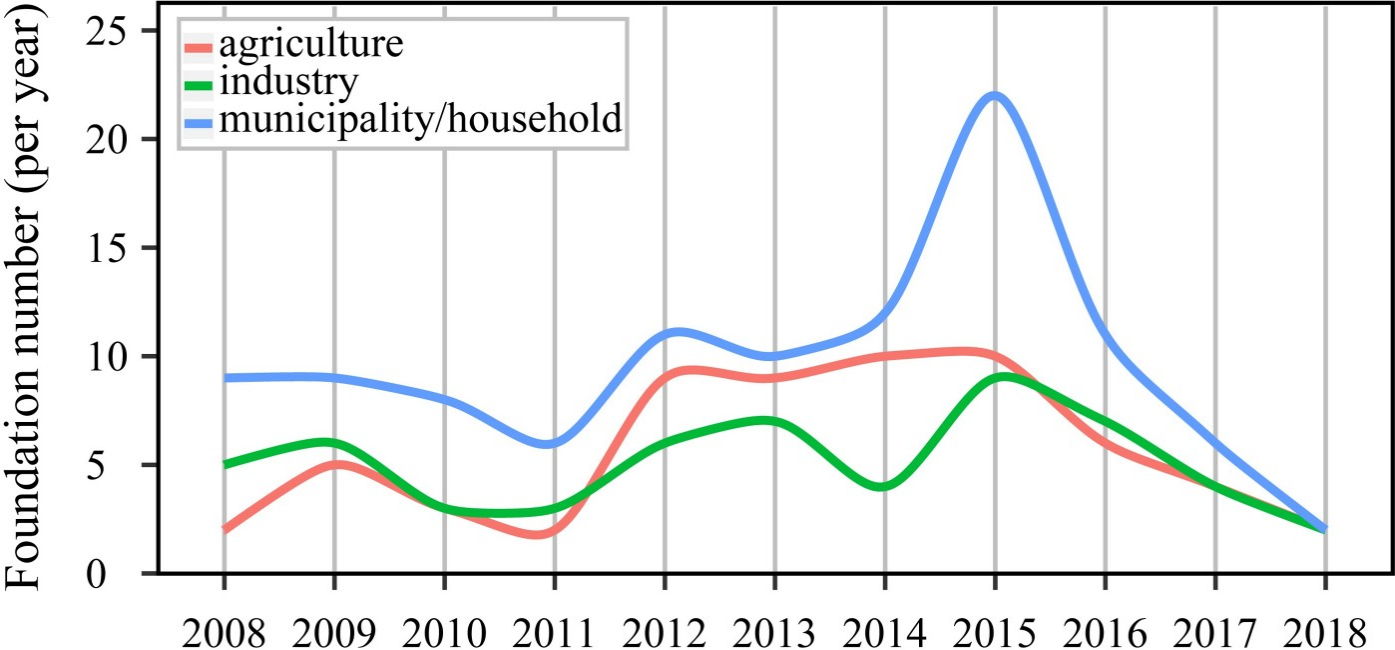
Changes in water startups numbers with different target markets from 2008 to 2018.

The municipal/household area occupied the most substantial part of the target market (Fig 2) over the entire period from 2008 to 2018 (Fig 3). The growth of population and urbanization could increase water demand and aggregate water pollution [27]. The urban residents also became more concerned about water safety in daily life as living standards improved, promoting the development of emerging water markets. Overall, the agricultural area constituted the second largest part of the target market (Fig 2). Particularly between 2012 and 2015, the number of water startups serviced in agricultural areas exceeded that in industrial areas (Fig 3). California ranks as the leading agricultural state nationwide based on the value of agricultural sales and production [28], and agriculture has a significant impact on water use in California. California has the highest number of irrigated farm acres and the highest water use per acre than other states in the US. This accounts for roughly 40%-80% of total water supplies based on different survey methods and assumptions [28]. More than 400 agricultural commodities were grown or produced in California [28], with an increasing shift to high-value, more permanent, and more water-intensive crops driven by market benefits [19]. For example, almonds, the state’s largest agricultural export by value [29], occupied 31% of the irrigated acres and had the second-largest net water use [28]. The high water consumption has promoted innovations in agriculture to increase water efficiency.

**Fig 4:**
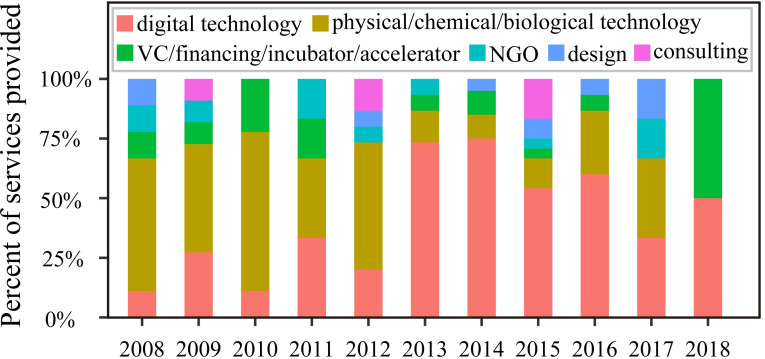
Changes in water startup numbers with different services from 2008 to 2018.

Digital technology was the most popular service provided by water startups, followed by physical/chemical/biological technology (Fig 2). There was no apparent difference between the services provided by NGO, consulting, design, and VC/financing/incubator/accelerator (Fig 2). Digital technology has increased dramatically since 2013, and has become more popular than other services (Fig 4). The popularity might be associated with the fast development of digital technology such as sensors [10] and big data analytics [11], which have brought about technological updates in the water industry. Undoubtedly, the application of digital technology has many potential advantages for the water area. For utilities, digital technology can monitor infrastructure's status to extend the operational life and reduce the overall cost of projects via the proactive replacement of hardware and/or software [30]. It can also help maintain close relationships with consumers by providing payment reminders, which would reduce the number of delinquent accounts and increase cash flow at the utility end. The real-time interactive digital techniques also assess quick customer feedback and help the water startups provide immediate improvements. On the other hand, the consumers can be alerted to high water consumption or leaks in real-time, reducing water bills and waste [30]. Such real-time management could increase public awareness of water use and further change social behavior. In addition, drinking water safety and public health could be secured via real-time water quality monitoring with advances in digital technology [30]. Therefore, digital water startups could be helpful for overall social benefits in a long term from the perspectives of economic costs, water-saving, and public health. For example, a program of real-time water meter-reading carried out by Mackay Regional Council, Queensland, Australia, from 2010 onwards, helped the utility defer $100M infrastructure costs for four years [31]. It also saved $20M net present value (NPV) by reducing 10% monthly peak demand, and improved customer knowledge and relations.

Physical/chemical/biological technology still plays an essential role in conventional water treatment (Figs 2 and 4). Particularly, due to the increasing demand on the effective removal of trace levels of emerging contaminants, it provides huge driving forces on upgrading the water infrastructures with advanced techniques. Among innovations applied in physical/chemical/biological technology, solar energy [9] and nanotechnology [8,32] enhanced techniques are the most promising ones. Solar technology's major engagement in water treatment is to power the desalination, detoxification, and disinfection with solar energy [9]. For example, a solar company (founded in 2011 in Palo Alto, California) is developing technologies for small and medium-scale solar thermal water heaters, solar water purification, and desalinization that avoid the use of costly materials and intricate manufacturing processes. The solar company has initiated projects for remote communities and coastal towns. On the other hand, nanotechnology and engineered nanomaterials were increasingly applied for water purification and wastewater treatment, including water quality monitoring, specialty adsorbents, high-performance membranes, and disinfection, etc. [8,32]. The application of nanotechnology could not only enhance treatment efficiency, but also promote the de-centralized water facilities, which would alleviate the risk of secondary contamination during the distribution process. For example, a company founded in 2009 in Concord, California, produces water purification cartridges for a point-of-use system to produce clean water that meets US EPA drinking water standards. Nano-fiber membranes are used to eliminate bacteria, protozoan cysts, and other contaminants. The water purification cartridge exhibits high performance with the feature of little maintenance and easy replacement.

### Water startup business performance and market feedback

**Fig 5:**
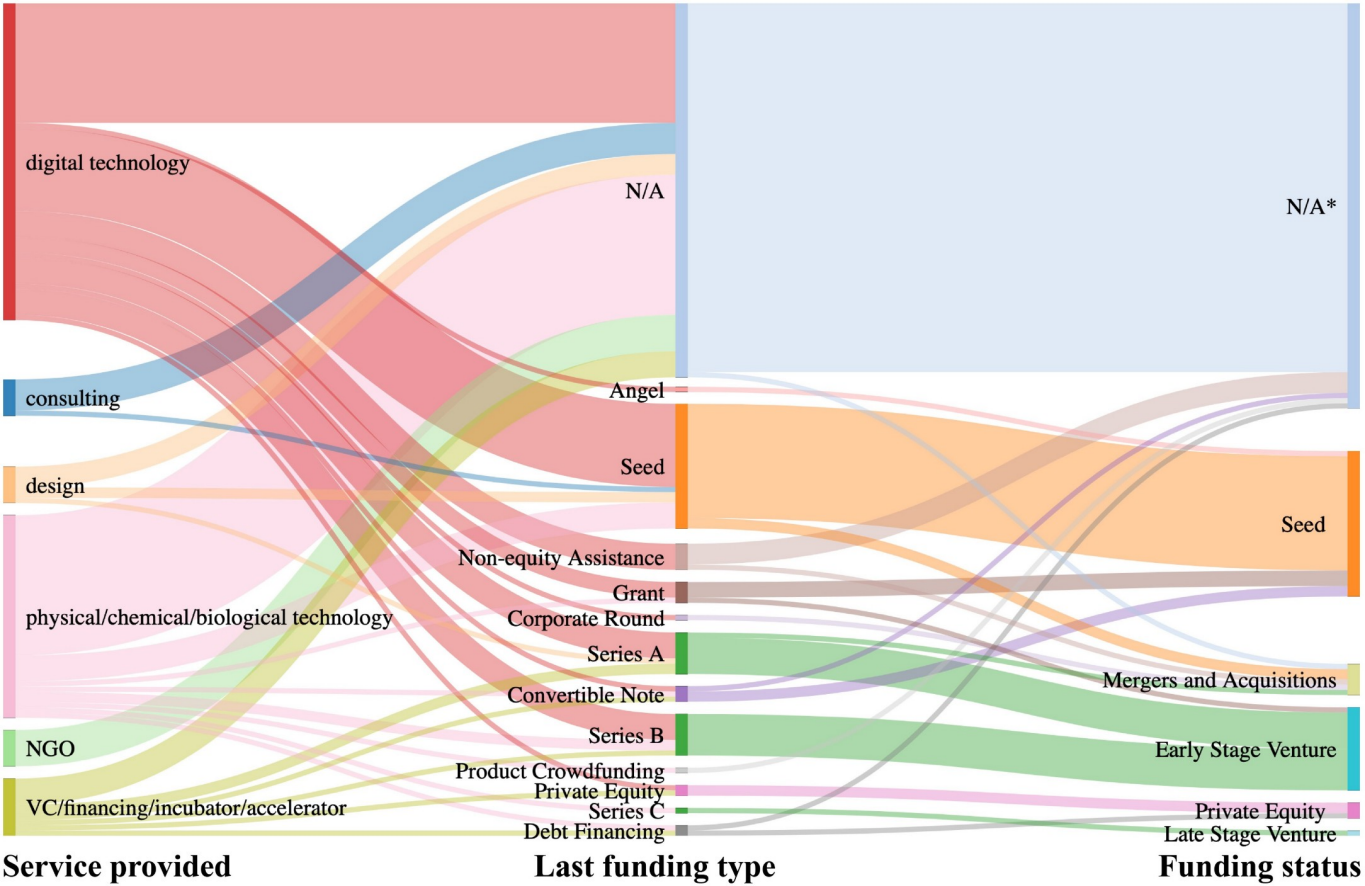
The Sankey diagram of water startups’ funding stages. * N/A means data not available.

A typical funding process includes the following stages: seed, early-stage venture, private equity, late-stage venture, and initial public offering (IPO) or acquisition. Most of the investigated water startups were still at the funding status of seed or early-stage ventures. Only four startups were in the funding stage of private equity or late-stage venture. All of the investigated startups were still far from the IPO stage according to theories of investment stages (Fig 5) [33–35]. The relatively slow development resulted from the following three reasons. First, major causes of water scarcity, such as population growth, take decades to happen [36], which would also lead to a delay in the response of market and investment. Second, investors would evaluate the political/regulatory risk, which usually involves numerous stakeholders with different interests and may compete for favorable regulations [37]. Political risk arises when politicians override the terms of agreed contracts, which creates barriers for water startups to raise money during different investment stages. Third, water companies are exposed to high-level liquidity risk as most water projects have a long duration of about 25–30 years [37], which would lead to the situation that investments are often long-lived and cannot be readily reversed and converted into cash. Therefore, the revenues and profits of water startups cannot meet the requirements for IPO in a short time.

The mean and median values of the total funding amounts and total revenues per year were used to evaluate the responses of investors to water startups and startups’ financial situations (Table 1). As the nature of VC/financing/incubator/accelerator is to raise money for investment, it had the highest mean and median value of total funding (Table 1). Digital technology ranked second with a mean value of $14.78 million, which indicates that investors were willing to invest in trending digital technology. For example, a digital water startup providing service for agriculture, raised $226 million (details in S1 Dataset). However, the median value of total funding for digital water startups was only $2.88 million, suggesting a significant variance in funding among digital water startups. Although digital technology was developing fast, not all digital water startups could get large amounts of funding. The digital services had a lower mean median value of total revenue per year (Table 1), which could be associated with the nature of lower purchasing frequency compared to other industries (e.g., physical/chemical/biological technology). Besides, most digital startups were founded after 2013 (Fig 4) and currently in the early development stage (Fig 5), resulting in a relatively smaller number of customers, which could be another reason for the lower revenue.

**Table 1:** Mean and median values (millions of dollars) of total capital funding and revenue for water startups providing different services^1^.

		VC/financing/incubator/accelerator	digital technology	physical/chemical/biological technology	Consulting^2^	Design^2^	NGO^2^
**Total capital funding (million dollars)**	mean	166.80	14.78	9.25	8.60	5.00	N/A^3^
	median	75.00	2.88	5.95	8.60	5.00	N/A^3^
**Kruskal-Wallis test**		Chi-square = 12.462; DF = 4; P = 0.0142					
**Revenue (million dollars/year)**	mean	2.53	3.77	8.13	7.18	3.00	4.84
	median	1.75	2.69	4.57	7.18	3.00	5.10
**Kruskal-Wallis test**		Chi-square = 6.207; DF = 5; P = 0.2866					

^1^Values were calculated from S1 Dataset.

^2^Consulting, NGO, and design categories only have 1–3 data points.

^3^N/A means that NGO does not have data on total capital funding.

**Fig 6:**
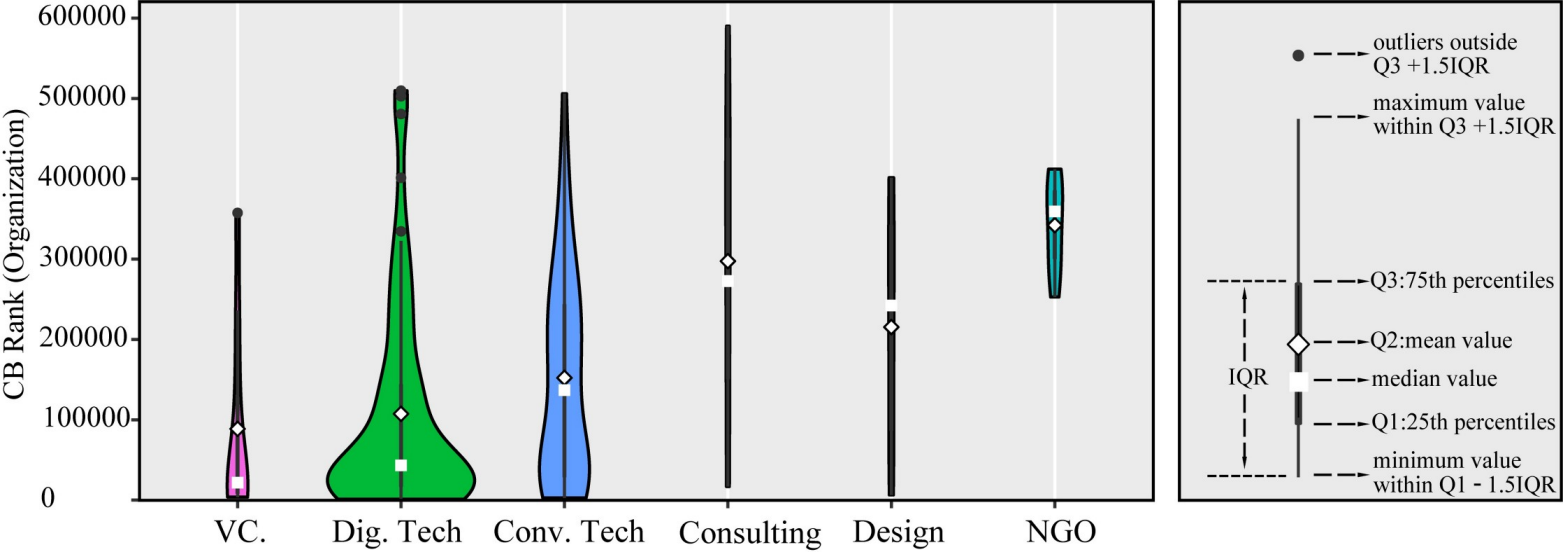
The CB Ranks of all water startups providing different services. VC.: VC/financing/incubator/accelerator. Dig. Tech: Digital technology. Conv. Tech: Physical/chemical/biological technology.

However, the fact that digital startups occupied the second place in the mean and median values of CB Rank (Fig 6) demonstrates their high potential in the market (The CB Rank is a dynamic ranking of all the entities in the Crunchbase dataset and measures the prominence of an entity. The smaller the CB Rank, the more prominent the organization. More information about CB Rank is provided in S3 Text). Note that digital technology may be used by homeowners more widely in the future, which would help construct smart water grids and increase overall social benefits in the long term. For example, a company was founded in 2015 in Los Angeles, California, and its business is to protect the entire homes from water damage and leaks. Consumers can get alerts on their phones in real-time if a leak is detected in the home, and can also set water consumption goals, monitor daily usage, and turn the water on and off remotely. The company declares that 17 gallons of water are lost every day due to leaks in a typical home, but 60% of homeowners had immediately discovered a leak with their service, saving 6205 gallons of water per year for each homeowner.

Physical/chemical/biological technology ranked medium in the mean value and median value of the total funding amount (Table 1) and CB Rank (Fig 6). However, the mean total revenue per year of the physical/chemical/biological technology was the highest at around $8.13 million per year because it is the leading technology for water treatment and has been widely accepted and used by the public. Also, it is characterized by high consumption frequency as consumers consume the chemical and/or biological materials continuously and need to maintain physical equipment regularly, which could increase the revenue.

To study consumer response, we evaluated the mean values of average monthly visits to sites (over the latest 6-month, and data was accessed from Crunchbase in February 2019). Furthermore, monthly page views per visit, bounce rate, and visit duration per visit (accessed from Crunchbase in February 2019). Detailed results were listed as S1 Table, with supplementary explanations of the indicators in S3 Text. Consulting and VC/financing/incubator/accelerator were more popular with consumers than other services based on overall levels of the four indicators.

### Business features and trends of digital water startups

**Fig 7:**
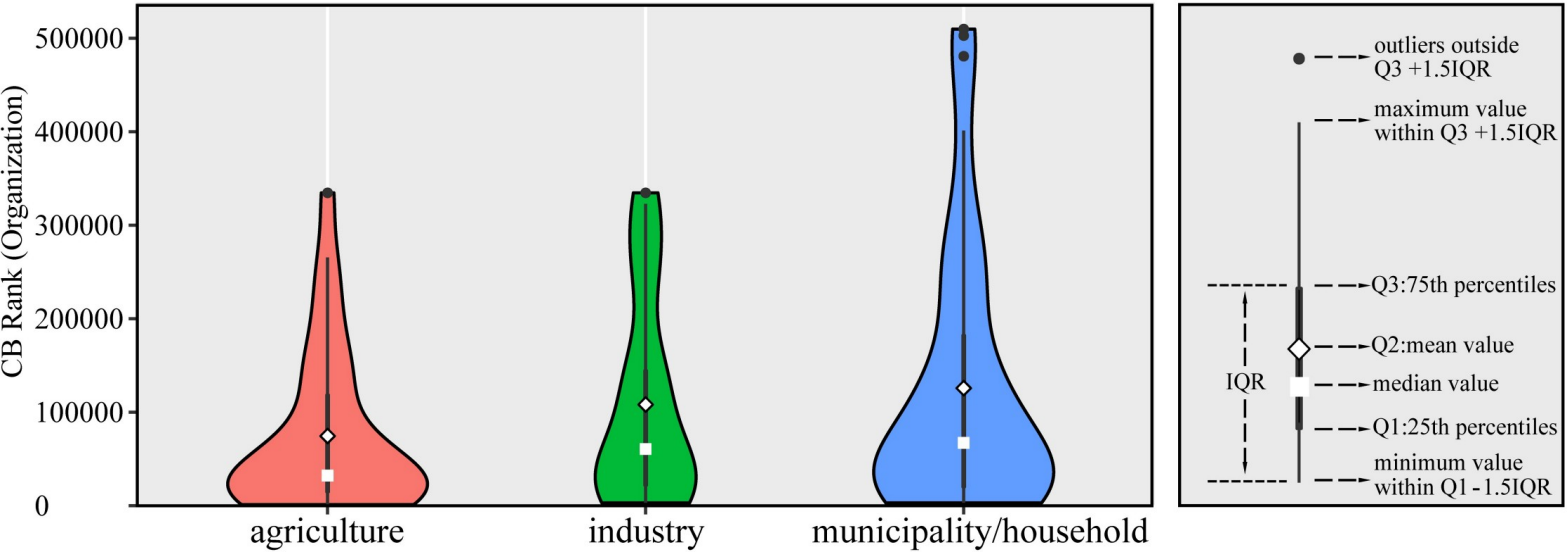
The CB Ranks of digital water startups with different target markets.

The preliminary analysis showed that digital technology exhibited high promise and long-term development trends. Further investigation was done on the capital, revenues, and consumer responses of digital technology. The primary customer of digital water startups was municipal/household area (45.26% of the customers), followed by agricultural and industrial areas (Table 2). The digital water startups serving the agricultural area raised the highest funding amount, averaging $18.16 million (Table 2). The agricultural area ranked lowest in terms of mean and median of CB rank (Fig 7), which indicates the prominence of digital technology in this area. But the mean and median total revenue of digital technology in agriculture was the lowest (Table 2). The relatively steep learning curve of adopting emerging technologies may be the major barrier since farmers have fewer motivations whenever the shifting process is complex, time-consuming, or expensive [38]. However, various subsidy and grant programs from the U.S. Department of Agriculture (USDA) and California Department of Food & Agriculture (CDFA) to support agricultural water development are available. For example, the State Water Efficiency and Enhancement Program (SWEEP) [39] provides financial assistance in the form of grants to implement irrigation systems that reduce greenhouse gases and save water across California agricultural operations. The CDFA has granted awards to 725 projects covering over 127,100 acres. $72.2 million has been awarded in the period up to and including 2019, with more than $47.7 million in matching funds contributed by awardees. Digital technology in the industrial area received higher mean and median total revenue compared with municipal/household and agricultural areas (Table 2), as the industries are usually at the frontier to adopt the advanced technologies to improve efficiency.

**Table 2:** Mean and median values (millions of dollars) of total capital funding and revenue, and percentages of digital water startups with different target markets^1^.

		agricultural area^2^	municipal/household area^2^	industrial area^2^
**Percentage**		31.58%	45.26%	23.16%
**Total capital funding (million dollars)**	mean	18.16	9.41	8.89
	median	2.96	1.60	2.92
**Kruskal-Wallis test**		Chi-square = 0.478; DF = 2; P = 0.7874		
**Revenue** **(million dollars/year)**	mean	2.99	4.41	5.90
	median	2.69	3.00	3.62
**Kruskal-Wallis test**		Chi-square = 1.242; DF = 2; P = 0.5374		

^1^Values were calculated from S1 Dataset.

^2^Some water startups that serve more than one area were counted more than once.

We evaluated the same indicators for the consumer response to digital water companies as that for all water startups (S2 Table), including the mean values of average monthly visits to sites (over the latest 6-month, and data was accessed from Crunchbase in February 2019). Furthermore, monthly page views per visit, bounce rate, and visit duration per visit (accessed from Crunchbase in February 2019). However, there were no apparent differences among the three service areas regarding customer response.

### Future perspectives and limitations

Population growth and urbanization are common factors intensifying water demand in many cities, but different cities have specific water problems based on other local situations. For example, high agricultural water usage and recent local wildfires in California increased water demand, while the main problem facing industrial cities around the Liao River Delta in China is water pollution [40]. Thus, providing solutions to local water issues should be the top priority for water startups. Meanwhile, the government should address more on foreseeing the potential water crisis in a mid-and-long term, and be proactive in water management to avoid catastrophic water shortages like the one experienced by Cape Town, South Africa [41]. Financial support from governments is also an essential source for those water startups with less revenue, for example, startups that use digital technology to serve the agriculture area. Furthermore, for entrepreneurs and scientists, to maximize the advantages of different sectors, it would be beneficial to engage digital technology and other emerging technologies into the conventional physical/chemical/biological technologies.

Notably, due to the data availability, some results may not be representative, for example, there were only 1–3 data points for the total capital funding and revenue in consulting, NGO, and design categories. Another major limitation lies on the limited sample size and short duration of available data to evaluate the consumer response, though web page visits may not necessarily result in actual customer relationships. A better way to evaluate consumers’ feedback is to conduct customer surveys, which should be investigated in future studies. Nevertheless, the main conclusions of current study are sound with concrete support of the presented data and analysis.

## Conclusions

In this study, we investigated the shifting landscape and development performance of emerging water startups in California. With population growth, urbanization, and improvements in living standards, most water startups chose to serve municipal/household areas. Agriculture was the second-largest target market for water startups due to California’s agricultural characteristics. An increasing fraction of the services provided were changing from conventional technologies to digital technologies. Investors were more interested in investing in digital water startups than in other services, but not all digital startups can get large amounts of funding. Although digital startups had the lowest mean total revenue, they could yield overall social benefits by decreasing water bills, increasing public awareness of water use, and protecting public health. Conventional physical/chemical/biological technologies were still crucial in emerging water markets, with higher revenues than other services, but the number of startups in this sector decreased. Consulting and VC/financing/incubator/accelerator were more popular with consumers than other services. These findings shed light on current water market conditions in California, and can be used to guide entrepreneurs, scientists, policymakers, and venture capitalists. Entrepreneurs and scientists would have better scenes of the technologies and service demand from the water markets, while the venture capitalists would improve the investment strategies based on the current overall performance of water startups. It also provides a solid reference for policymakers to formulate relevant measures and provide financial support to promote the sustainable development of emerging water markets.

## Supporting information

S1 Table Consumer response for overall water startups. (DOC)

S2 Table Consumer response for digital water startups. (DOC)

S1 Dataset The full list of water startups. (XLSX)

S1 Text The Search strategy of data collection. (DOC)

S2 Text Services provided by the water startups. (DOC)

S3 Text Evaluation indicators. (DOC)
