# Supplementary material for: Shifting entrepreneurial landscape and development performance of water startups in emerging water markets
Source: PLoS One. 2021 Feb 4;16(2):e0246282. doi: 10.1371/journal.pone.0246282 (PMC7861426; doi:10.1371/journal.pone.0246282)
Supplement: S1 Table — (DOC) [file pone.0246282.s001.doc]

**Supporting Information**

**for**

**Shifting entrepreneurial landscape and development performance of water startups in emerging water markets**

Peiyuan Liu^1^, Yuxiong Huang^1*^, Slav W. Hermanowicz^1,2^

^1^ Tsinghua-Berkeley Shenzhen Institute, Tsinghua Shenzhen International Graduate School, Tsinghua University, Shenzhen, China
^2^ Department of Civil and Environmental Engineering, University of California, Berkeley, CA, United States

^*^ Corresponding author

E-mail: [huang_yuxiong@sz.tsinghua.edu.cn](mailto:huang_yuxiong@sz.tsinghua.edu.cn)

**S1 Table. Consumer response for overall water startups.**

|  | VC. | Consulting | Design | Dig. Tech | Conv. Tech | NGO |
| --- | --- | --- | --- | --- | --- | --- |
| **mean of total average visits (6 months)** | 48071.10 | 8802.72 | 7168.51 | 4601.53 | 4220.28 | 3202.72 |
|  | Consulting | Conv. Tech | NGO | VC. | Dig. Tech | Design |
| **mean of total page views/visit (last month)** | 3.66 | 2.70 | 2.38 | 2.31 | 2.08 | 2.08 |
|  | Design | VC. | Dig. Tech | NGO | Consulting | Conv. Tech |
| **mean of total bounce rate** | 60.33% | 57.10% | 53.74% | 52.31% | 50.97% | 43.47% |
|  | Consulting | VC. | Conv. Tech | Design | Dig. Tech | NGO |
| **mean of total visit duration** | 219.00 | 189.57 | 143.63 | 112.67 | 109.36 | 88.20 |

VC.: VC/financing/incubator/accelerator. Dig. Tech: digital technology. Conv. Tech: physical/chemical/biological technology.
