# Supplementary material for: Shifting entrepreneurial landscape and development performance of water startups in emerging water markets
Source: PLoS One. 2021 Feb 4;16(2):e0246282. doi: 10.1371/journal.pone.0246282 (PMC7861426; doi:10.1371/journal.pone.0246282)
Supplement: S2 Table — (DOC) [file pone.0246282.s002.doc]

**Supporting Information**

**for**

**Shifting entrepreneurial landscape and development performance of water startups in emerging water markets**

Peiyuan Liu^1^, Yuxiong Huang^1*^, Slav W. Hermanowicz^1,2^

^1^ Tsinghua-Berkeley Shenzhen Institute, Tsinghua Shenzhen International Graduate School, Tsinghua University, Shenzhen, China
^2^ Department of Civil and Environmental Engineering, University of California, Berkeley, CA, United States

^*^ Corresponding author

E-mail: [huang_yuxiong@sz.tsinghua.edu.cn](mailto:huang_yuxiong@sz.tsinghua.edu.cn)

**S2 Table. Consumer response for digital water startups.**

|  | municipality/household | agriculture | industry |
| --- | --- | --- | --- |
| **mean of total average visits (6 months)** | 4853.89 | 4541.62 | 4356.57 |
|  | agriculture | industry | municipality/household |
| **mean of total page views/visit (last month)** | 2.34 | 2.23 | 2.10 |
|  | industry | municipality/household | agriculture |
| **mean of total bounce rate** | 58.77% | 56.07% | 54.13% |
|  | agriculture | industry | municipality/household |
| **mean of total visit duration** | 116.45 | 112.92 | 112.29 |
