# Supplementary material for: Shifting entrepreneurial landscape and development performance of water startups in emerging water markets
Source: PLoS One. 2021 Feb 4;16(2):e0246282. doi: 10.1371/journal.pone.0246282 (PMC7861426; doi:10.1371/journal.pone.0246282)
Supplement: S1 Text — (DOC) [file pone.0246282.s004.doc]

**Supporting Information**

**for**

**Shifting entrepreneurial landscape and development performance of water startups in emerging water markets**

Peiyuan Liu^1^, Yuxiong Huang^1*^, Slav W. Hermanowicz^1,2^

^1^ Tsinghua-Berkeley Shenzhen Institute, Tsinghua Shenzhen International Graduate School, Tsinghua University, Shenzhen, China
^2^ Department of Civil and Environmental Engineering, University of California, Berkeley, CA, United States

^*^ Corresponding author

E-mail: [huang_yuxiong@sz.tsinghua.edu.cn](mailto:huang_yuxiong@sz.tsinghua.edu.cn)

**S1 Text. The Search strategy of data collection.**

The Crunchbase’s company filters were used to retrieve relevant water companies by searching keywords: headquarters location (California), description (water), and founded date (between 1/1/2008 and 12/31/2018), resulting in 269 water startups.

Furthermore, we supplemented data from another two platforms, AngelList and Mattermark, to ensure comprehensive data collection. On the AngelList platform, search keywords were location (California) with market (water/water purification) or tech (water technology/ water purification/ waterproof/ waterjet), resulting in 128 water startups. Since AngelList does not have the search condition of founded date, thus we eliminated those water startups not founded between 2008 and 2018 in the process of calibration. On the Mattermark platform, we used the advanced filter to search: state (California), founded (greater than or equal to 2008), keywords (water), and description (water), resulting in 129 water startups. By this way, we got another 257 water startups, with 128 from AngelList and 129 from Mattermark. We further verified the supplemented 257 startups on Crunchbase. Eventually, 46 of them were supplemented, with 25 from AngelList and 21 from Mattermark.

We calibrated data accuracy of 315 water startups on Crunchbase. We verified their locations (California), founding date (between 2008 and 2018), and specific services provided (improving water quality, saving water quantity, or increasing water management efficiency) by checking their official websites and Crunchbase. The startups whose location, founding date and specific services do not meet the requirement were deleted. Finally, we got 132 water startups on the list for the further analysis.
