# Supplementary material for: Shifting entrepreneurial landscape and development performance of water startups in emerging water markets
Source: PLoS One. 2021 Feb 4;16(2):e0246282. doi: 10.1371/journal.pone.0246282 (PMC7861426; doi:10.1371/journal.pone.0246282)
Supplement: S2 Text — (DOC) [file pone.0246282.s005.doc]

**Supporting Information**

**for**

**Shifting** **entrepreneurial landscape and development performance of water startups in emerging water markets**

Peiyuan Liu^1^, Yuxiong Huang^1*^, Slav W. Hermanowicz^1,2^

^1^ Tsinghua-Berkeley Shenzhen Institute, Tsinghua Shenzhen International Graduate School, Tsinghua University, Shenzhen, China
^2^ Department of Civil and Environmental Engineering, University of California, Berkeley, CA, United States

^*^ Corresponding author

E-mail: [huang_yuxiong@sz.tsinghua.edu.cn](mailto:huang_yuxiong@sz.tsinghua.edu.cn)

**S2 Text. Services provided by the water startups.**

Services provided include design, consulting, Venture Capital (VC)/financing/incubator/accelerator, Non-Governmental Organization (NGO) activities, digital technology, and physical/chemical/biological technology. Specifically, design startups help design sustainable construction, landscaping, or home improvement with higher water efficiency. Consulting startups offer relevant water strategies and recommendations to government, private sectors, and homeowners. Venture Capital firms invest in water startups at a variety of stages, ranging from seed to Series A and beyond; some finance companies provide financing service for energy, water, and renewable energy projects for household or commercial use; incubator and accelerator have fixed-term on-site programs for water startups and take a set amount of seed equity in exchange for capital and mentorship, connections, etc. NGO provides technology, training, or relevant media publicity to help people save water, improve water quality, and raise public awareness of water use. Digital technologies include sensors, big data analytics, drones, etc. Physical/chemical/biological technologies include nano-enabled membranes, solar-enabled desalination, bioreactor, agrochemicals, and waterless car cleaning technology, etc.
