# Supplementary material for: Shifting entrepreneurial landscape and development performance of water startups in emerging water markets
Source: PLoS One. 2021 Feb 4;16(2):e0246282. doi: 10.1371/journal.pone.0246282 (PMC7861426; doi:10.1371/journal.pone.0246282)
Supplement: S3 Text — (DOC) [file pone.0246282.s006.doc]

**Supporting Information**

**for**

**Shifting entrepreneurial landscape and development performance of water startups in emerging water markets**

Peiyuan Liu^1^, Yuxiong Huang^1*^, Slav W. Hermanowicz^1,2^

^1^ Tsinghua-Berkeley Shenzhen Institute, Tsinghua Shenzhen International Graduate School, Tsinghua University, Shenzhen, China
^2^ Department of Civil and Environmental Engineering, University of California, Berkeley, CA, United States

^*^ Corresponding author

E-mail: [huang_yuxiong@sz.tsinghua.edu.cn](mailto:huang_yuxiong@sz.tsinghua.edu.cn)

**S3 Text. Evaluation indicators.**

1. Funding status: describes the organization’s most recent funding status, such as early stage venture, late stage venture, Mergers, and Acquisitions (M&A).
2. Funding types: includes Angel, Pre-Seed, Seed, Series A, Series B, Series C, Private Equity, Convertible Note, Debt Financing, Secondary Market, Grant, Corporate Round, etc. [1].
3. Total funding amount: the funding raised across all funding rounds.
4. Revenue: the revenue a company generates per year. For private companies, it is an estimate.
5. CB Rank: a dynamic ranking for all entities (i.e., companies, organizations, and schools) in the Crunchbase dataset. It measures the prominence of an entity. It is determined from an algorithm looking at total funding amount, page views, follows, funding events, news articles, acquisitions, the number of connections a profile has, the level of community engagement, and more. A company's CB Rank is fluid and subject to rising and decaying over time with time-sensitive events. Events such as product launches, funding events, leadership changes, and news affect a company's CB Rank [2–4].
6. Average visits: the average of monthly visits to site over the past 6 months before accessing the database (February 2019), including both desktop and mobile web [5].
7. Page views/visit: the average number of pages viewed by users in each visit to a site in the last month before accessing the database (February 2019), including desktop and mobile web.
8. Bounce rate: the percentage of visitors to site who navigate away after viewing only one page, including both desktop and mobile web.
9. Visit duration: the average time spent by users on a website per visit in seconds, including both desktop and mobile web.

**References:**

1. Crunchbase Staff. Glossary of Funding Types. 2019. Available: https://support.crunchbase.com/hc/en-us/articles/115010458467-Glossary-of-Funding-Types

2. Crunchbase Staff. Crunchbase Rank (CB Rank). 2019. Available: https://support.crunchbase.com/hc/en-us/articles/115010477187-Crunchbase-Rank-CB-Rank

3. Schools D. How to Use Crunchbase Rank & Trend Score to Find Influential Companies & Market Trends. 2016. Available: https://about.crunchbase.com/blog/influential-companies/

4. Stephan D. What Is Crunchbase Rank and Trend Score? 2019. Available: https://about.crunchbase.com/blog/crunchbase-rank-trend-score/

5. Crunchbase Staff. How to use SimilarWeb on Crunchbase Profiles. 2019. Available: https://support.crunchbase.com/hc/en-us/articles/115015423647-How-to-use-SimilarWeb-on-Crunchbase-Profiles
